# Causal Effect of Selenium Levels on Osteoporosis: A Mendelian Randomization Study

**DOI:** 10.3390/nu15245065

**Published:** 2023-12-11

**Authors:** Jinjie Li, Hong Li, Amin Ullah, Shuyuan Yao, Quanjun Lyu, Guangning Kou

**Affiliations:** 1 Centre for Nutritional Ecology and Centre for Sport Nutrition and Health, Zhengzhou University, Zhengzhou 450001, China; 2 Department of Nutrition and Food Hygiene, School of Public Health, Zhengzhou University, Zhengzhou 450001, China

**Keywords:** Mendelian randomization, causal association, trace element, selenium, osteoporosis

## Abstract

Prior research has demonstrated equivocal associations between selenium (Se) concentrations and osteoporosis (OP), yielding inconclusive findings. The purpose of the current study was to examine the potential correlation between Se levels and the risk of OP by using the Mendelian randomization (MR) study design. The genetic variants related to Se levels were obtained from a meta-analysis of a Genome-Wide Association Study (GWAS) conducted on toenail Se levels (*n* = 4162) and blood Se levels (*n* = 5477). The data summary for OP and bone mineral density (BMD) was obtained by utilizing the GWAS database. To examine the association between Se levels and BMD and OP, we employed three statistical methods: inverse variance weighted, weighted median, and MR-Egger. The reliability of the analysis was verified by sensitivity testing. All three methods of MR analysis revealed that Se levels had no effect on OP risk. In addition, the sensitivity analysis revealed no heterogeneity or pleiotropy, and the significance of the overall effect remained unaffected by single-nucleotide polymorphisms (SNPs), as determined by the leave-one-out analysis, indicating that our findings are relatively reliable. The results of our study indicate that there is no causal association between Se levels and the risk of OP. However, additional investigation is necessary to ascertain whether there is a potential association between these variables.

## 1. Introduction

As the world’s population ages, chronic metabolic diseases are becoming more prevalent, posing a significant public health concern on a global scale [1,2]. Osteoporosis (OP) is one of the most prevalent skeletal disorders. In the 2010 U.S. Census, there were approximately 10.2 million individuals over the age of 50 with osteoporosis, and the percentage of women with osteoporosis was much higher (16.5%) than men (5.1%) [3,4]. The risk of fracture increases significantly with the onset of OP [5]. The occurrence of a fracture brings with it a series of burdens, including reduced quality of life, increased financial burden, and significant psychological consequences. Especially in the elderly, a major fracture has a high probability of leading to death within a year [6]. Reduced bone mineral density (BMD) and consequent bone loss are the defining features of OP [7,8,9]. The lumbar spine (LS) and femoral neck (FN) BMDs are the most commonly used and genetically related indicators for assessing OP [4,10,11]. The onset of OP is usually associated with factors such as genetics, age, malnutrition, endocrine disruption and obesity [12,13,14]. In addition, some genetic mutations may also increase the risk of OP [15]. It is challenging to detect OP in its earlier stages, and many uncertain potential risks could have a significant impact on patients. Therefore, it is necessary to investigate the causes of OP and implement effective preventative measures.

Selenium (Se) is an essential trace element with significant health benefits [16,17]. Humans obtain it primarily through their diet or nutritional supplements [18]. Selenoproteins, the predominant form of Se, play a key role in anti-inflammation, neuroprotection, and inhibition of oxidative stress [19,20,21]. However, deficiency of Se in the body or excessive intake can also be harmful [22,23,24,25,26]. The association between Se and OP has been investigated for several years; however, the results still need to be clarified. Some studies have demonstrated a negative correlation between Se and the risk of OP [27,28,29], whereas others have shown no association between Se and OP [30,31,32]. Given the inconclusive nature of the association between Se and OP and the potential influence of confounding variables in previous studies, further research is required to determine their association.

Mendelian randomization (MR) is a genetic variation-based method for inferring the causal association between exposures and outcomes [33]. There are several requirements that must be met when instrumental variables (IVs) are used as proxies for target exposures. In general, the genetic instrument chosen must be highly correlated with the exposure, and only then can the genetic instrument logically substitute for the exposure. Diseases typically have a number of risk factors, and it is uncertain whether the substitution of the exposure with a genetic IV directly influences the outcome or whether it first affects the risk factors and is thus indirectly related to the outcome. Therefore, it is also necessary to eliminate IVs that may be associated with the risk factors. In addition, to ensure that the outcome is indeed caused by the exposure factor, there must be no association between the IV and the outcome [34]. According to Mendel’s laws of heredity, genetic variation is random, determined before birth and not altered by acquired factors [35]. Thus, MR studies are considered the natural genetic equivalent of randomized controlled experiments, eliminating confounding variables and reverse causality compared to traditional observational studies [36,37]. MR is now extensively employed to analyze the causal association between exposure and disease [38,39,40]. Therefore, we intended to determine the association of Se levels with the risk of OP by performing MR analysis using publicly available GWAS data.

## 2. Materials and Methods

### 2.1. Study Overview

**Figure 1:**
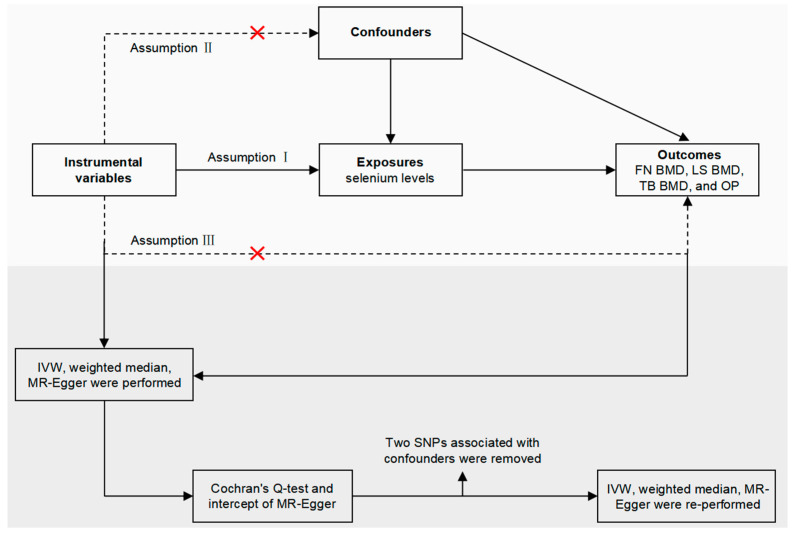
The three assumptions of Mendelian randomization studies and the analysis process of this study. FN BMD: femoral neck bone mineral density; LS BMD: lumbar spine bone mineral density; TB BMD: total body bone mineral density; OP: osteoporosis; IVW: inverse variance weighted; SNPs: single-nucleotide polymorphisms.

The detailed analysis process for this study is shown in Figure 1. Toenail selenium (Se) and blood Se levels were selected as exposures, and femoral neck (FN) bone mineral density (BMD), lumbar spine (LS) BMD, total body (TB) BMD and osteoporosis (OP) were selected as outcomes. Summary data on exposures or outcomes were collected from published GWAS meta-analyses and publicly available data. These summary data were analyzed by Mendelian randomization (MR) to determine if there was a causal association between Se levels and the risk of OP. Our MR study meets three basic assumptions: (1) the selected IVs exhibit a strong association with the exposure factors; (2) the IVs are not associated with confounding variables that might influence the outcome and (3) the IVs can only impact the outcome via the exposure, and there is no direct relationship between the IVs and the outcome. Since this study utilized publicly accessible databases, no additional ethical approval was necessary. Sensitivity tests were also performed in this study using different methods to assess the reliability of the findings. No causal relationship was reported between Se levels and any of the LS BMD, FN BMD, TB BMD or OP, according to the final results.

### 2.2. Summary Data for Se Levels

Blood Se and toenail Se levels were used as genetic IVs for in vivo Se concentration in this study. An extensive GWAS meta-analysis of toenail Se and blood Se levels in European and Australian samples was used to obtain Se level-related single-nucleotide polymorphisms (SNPs) [41]. The GWAS meta-analysis of toenail Se levels included 4162 individuals from 4 different US cohorts (adjusted for gender, residence, smoking status and age) [41]; the GWAS meta-analysis of blood Se levels was performed on two cohorts, including 2874 pregnant women from the United Kingdom and 2603 twins and their families from Australia (adjusted for age, consanguinity and gender) [42]. Detailed information on the toenail Se and blood Se cohorts can be found in the studies by Cornelis et al. and Evans et al. [41,42].

### 2.3. Instrumental Variable Selection

To ensure the independent existence of each SNP, all SNPs associated with Se concentration (*p* < 5 × 10^−8^) were trimmed for linkage disequilibrium (r^2^ = 0.3) to remove highly correlated SNPs. For those SNPs that were missing in the outcome data, they were replaced by SNPs with r^2^ greater than 0.8. Similar to previous studies, 11 independent SNPs were identified that were strongly associated with Se levels [43]. Details on SNPs are shown in Table 1. In order to detect weak IV bias, the F-statistics of the SNPs were utilized; F > 10 signifies the absence of weak IV bias [44]. The formula for the F-statistic is as follows: F = R^2^ × (N − 2)/(1 − R^2^), where R^2^ is the degree of exposure explained by the IVs and N is the number of SNPs. The formula for R^2^ is as follows: R^2^ = [2 × Beta^2^ × (1 − EAF) × EAF]/[2 × Beta^2^ × (1 − EAF) × EAF + 2 × SE^2^ × N × (1 − EAF) × EAF], where Beta represents the genetic effect of the SNP, EAF (effect allele frequency), SE (standard error) and N the sample size. The F-statistics of all SNPs were calculated to be greater than 10, making them unbiased for predicting IVs and effective for predicting OP. After the initial analysis was completed, the SNPs associated with OP were checked using Phenoscanner V2. Finally, we found two SNPs that were potentially associated with OP incidence (*p* < 5 × 10^−8^). After removing the relevant SNPs, the MR analysis was repeated to validate the accuracy of our findings.

### 2.4. GWAS Summary Data for OP

By using FN BMD, LS BMD, TB BMD and OP, the causal effect of Se levels on OP risk was investigated. These data were obtained from publicly available databases (https://gwas.mrcieu.ac.uk/, accessed on 18 October 2023). The FN BMD (*n* = 32,735) and LS BMD (*n* = 28,498) data were derived from a whole-genome sequencing analysis of European participants [45]. The TB BMD summary data were derived from a GWAS meta-analysis in which participants were predominantly from the general European population (86%), with the remaining 14% participants from mixed backgrounds [46]. OP summary data were obtained from the IEU GWAS database, with a total of 484,598 participants from Europe, including 7751 cases and 476,847 controls [47]. Table 2 shows all GWAS data.

### 2.5. Mendelian Randomization Analyses

In order to increase the reliability of the study results, the causal relationship between Se concentrations and FN BMD, LS BMD, TB BMD and OP risk was investigated using three Mendelian randomization methods. IVW, which assumes that each genetic variant exists independently and can influence outcome only through the exposure of interest and combines the Wald ratios of individual SNPs, was employed as the principal method of analysis in this study. However, causality may be biased in the presence of pleiotropy [48,49]. The MR-Egger method allows genetic variants to have pleiotropic effects, but requires that pleiotropic effects be independent of variant–exposure relationships [48]. As long as more than half of the instruments used in the MR analysis are valid, the weighted median (WM) approach allows invalid instruments to be used and also allows variation to be related to confounders of the exposure–outcome relationship [49]. To obtain more precise analysis results, we used the WM and MR-Egger methods as complementary methods to IVW, although they are less powerful (wider CI) [50]. A statistically significant association between exposure and outcome was deemed to be present when the *p*-value was found to be less than 0.05.

### 2.6. Sensitivity Analysis

In the case of horizontal pleiotropy, which can occur when variants linked to Se levels influence outcomes via alternative pathways, the independence and exclusivity assumptions may be violated, ultimately resulting in unreliable MR results. Therefore, we examined the heterogeneity and pleiotropy of the results. Heterogeneity was evaluated using Cochran’s Q-test (*p* < 0.05 was considered heterogeneity), and pleiotropy was assessed through the MR-Egger intercept (*p* < 0.05 was considered pleiotropy) [51]. In addition, a leave-one-out (LOO) analysis was conducted. LOO analysis refers to the exclusion of a single SNP, followed by MR analysis, which is used to evaluate the impact of the SNP on the outcome. If the results change significantly after the SNP is removed, it indicates that the outcome is sensitive to the IVs [52]. In this study, R software and the TwoSampleMR package were used for all MR analyses.

## 3. Results

### 3.1. Effects of Se on FN BMD

**Figure 2:**
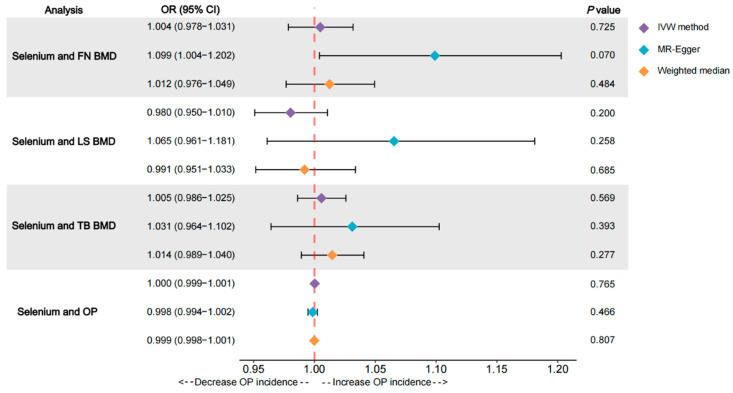
Odds ratio plot of selenium levels with OP and BMD. OR: odds ratio; FN BMD: femoral neck bone mineral density; LS BMD: lumbar spine bone mineral density; TB BMD: total body bone mineral density; OP: osteoporosis; IVW: inverse variance weighted.

**Figure 3:**
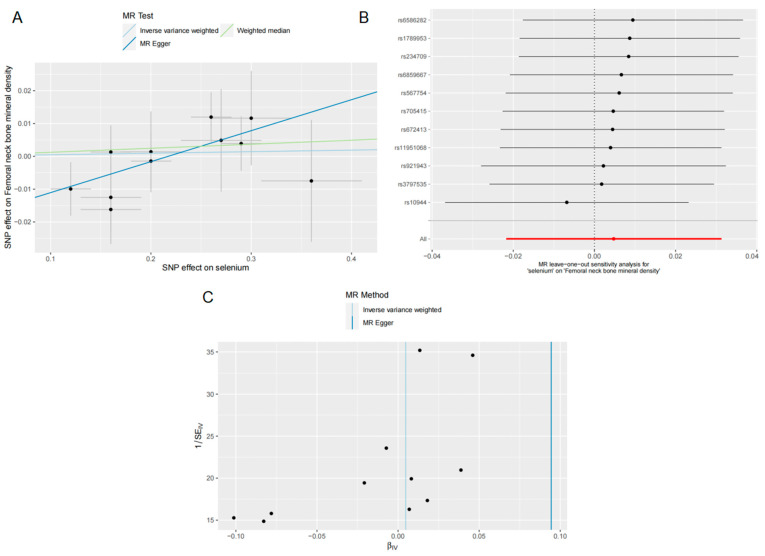
Effects of selenium on FN BMD. (**A**) Scatter plot of the causal effect of selenium concentrations on FN BMD; (**B**) forest plot of the LOO analysis; (**C**) funnel plot of the causal effect of selenium concentrations on FN BMD.

We did not observe a statistically significant causal relationship between Se concentrations and FN BMD using the IVW method (OR = 1.004, 95% CI = 0.978–1.031, *p* = 0.725) based on our analysis of the 11 Se level-related SNPs (refer to Figure 2). The WM (OR = 1.012, 95% CI = 0.976–1.049, *p* = 0.484) and MR-Egger regression (OR = 1.099, 95% CI = 1.004–1.202, *p* = 0.070) results were in agreement with the IVW (Figure 2). This lack of association was further supported by the scatter plot presented in Figure 3A. We then examined the heterogeneity and pleiotropy of the results. Cochran’s Q-test results for MR-Egger (Q = 4.919; *p* = 0.841) and IVW (Q = 9.058; *p* = 0.526) showed no heterogeneity in any of the analyses, and the MR-Egger intercept (intercept = −0.020; *p* = 0.072) analysis showed intercepts close to 0 (Table 3). The LOO analysis revealed that no individual SNP altered the overall effect (Figure 3B). Furthermore, the funnel plot was nearly symmetric (Figure 3C), showing the absence of pleiotropy.

### 3.2. Effects of Se on LS BMD

**Figure 4:**
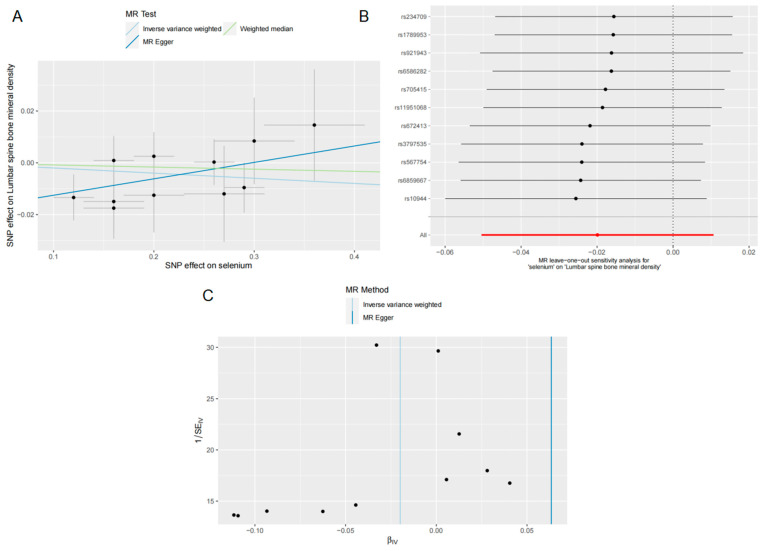
Effects of selenium on LS BMD. (**A**) Scatter plot of the causal effect of selenium levels on LS BMD; (**B**) Forest plot of the LOO analysis; (**C**) funnel plot of the causal effect of selenium levels on LS BMD.

For the relationship between Se levels and LS BMD, the IVW method failed to establish a causal relationship between Se levels and LS BMD (OR = 0.980, 95% CI = 0.950–1.010, *p* = 0.200) (Figure 2). The IVW result was confirmed by the WM (OR = 0.991, 95% CI = 0.951–1.033, *p* = 0.685) and MR-Egger (OR = 1.065, 95% CI = 0.961–1.181, *p* = 0.258) methods (Figure 2). This lack of association was further supported by the scatter plot shown in Figure 4A. For the test of heterogeneity, neither the MR-Egger (Q = 4.827; *p* = 0.849) nor the IVW (Q = 7.574; *p* = 0.670) Cochran’s Q test results showed statistical significance (Table 3). The absence of pleiotropy for the IVs was confirmed by the MR-Egger intercept analysis (intercept = −0.018; *p* = 0.131) for the pleiotropy test (Table 3). The LOO analysis shows that the overall result is not affected by excluding individual SNPs (Figure 4B), and the funnel plot is almost symmetrical (Figure 4C), meaning that our analysis is relatively robust.

### 3.3. Effects of Se on TB BMD

**Figure 5:**
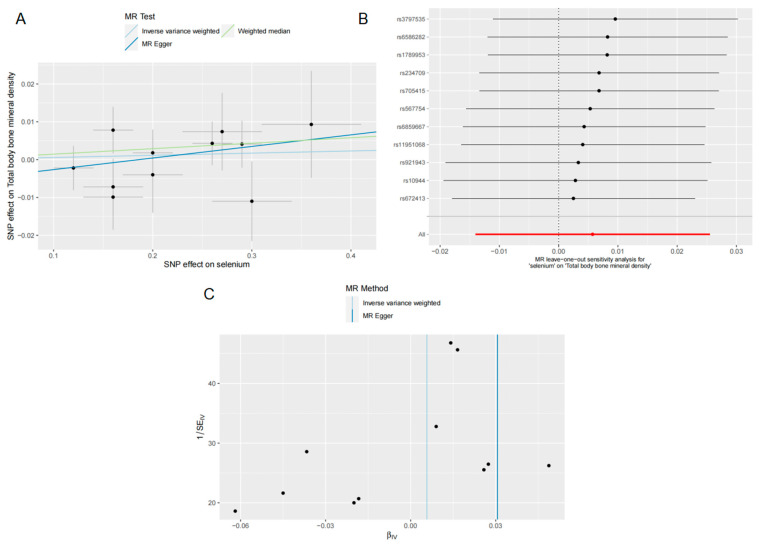
Effects of selenium on TB BMD. (**A**) Scatter plot of the causal effect of selenium levels on TB BMD; (**B**) forest plot of the LOO analysis; (**C**) funnel plot of the causal effect of selenium levels on TB BMD.

The IVW results (OR = 1.005, 95% CI = 0.986–1.025, *p* = 0.569) did not indicate a causal effect of Se levels on TB BMD in the analysis of TB BMD (Figure 2). As indicated by the results obtained from the WM (OR = 1.014, 95% CI = 0.989–1.040, *p* = 0.277) and MR-Egger (OR = 1.031, 95% CI = 0.964–1.102, *p* = 0.393) methods, there was no causal relationship identified between Se concentrations and TB BMD (Figure 2). This lack of association was further supported by the scatter plot presented in Figure 5A. When tested for heterogeneity, MR-Egger (Q = 6.460; *p* = 0.693) and IVW (Q = 7.040; *p* = 0.721) did not show statistical differences (Table 3), indicating that our IVs were stable. The LOO analysis showed that the overall effect was relatively stable, and the funnel plot was close to symmetrical (Figure 5B,C), which was confirmed by the MR-Egger intercept (intercept = −0.005; *p* = 0.465) analysis (Table 3). These results consistently demonstrated that our analyses were not biased.

### 3.4. Effects of Se on OP

**Figure 6:**
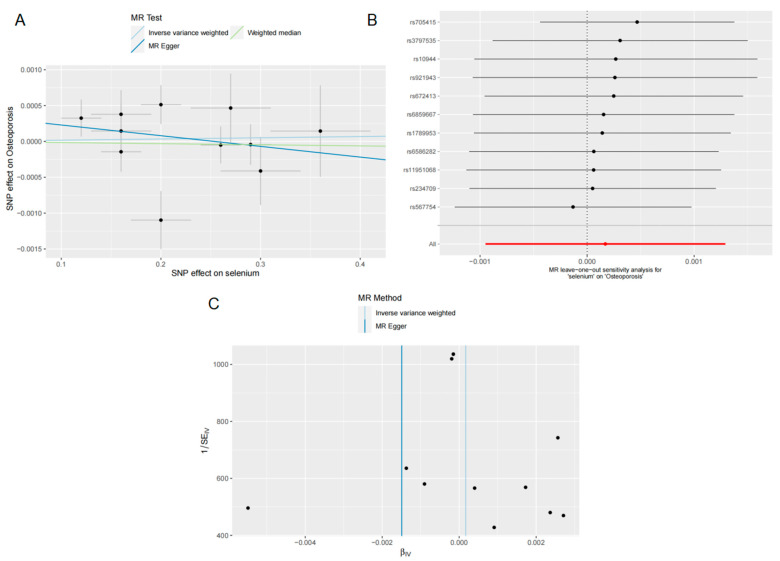
Effects of selenium on OP. (**A**) Scatter plot of the causal effect of selenium levels on OP; (**B**) forest plot of the LOO analysis; (**C**) funnel plot of the causal effect of selenium levels on OP.

Finally, we assessed the association between Se levels and OP risk by using MR analysis. In accordance with the findings of FN BMD, LS BMD and TB BMD, the IVW (OR = 1.000, 95% CI = 0.999–1.001, *p* = 0.765), MR-Egger regression (OR = 0.998, 95% CI = 0.994–1.002, *p* = 0.466) and WM methods (OR = 0.999, 95% CI = 0.998–1.001, *p* = 0.807) showed that Se levels were not significantly associated with OP risk (Figure 2). This lack of association was further supported by the scatter plot presented in Figure 6A. We then tested for heterogeneity, which was not observed in the Cochran’s Q-test results for MR-Egger (Q = 14.772; *p* = 0.097) and IVW (Q = 16.063; *p* = 0.097) (Table 3). The MR-Egger intercept (intercept = 0.000; *p* = 0.398) analysis showed no evidence of pleiotropy between Se levels and the risk of OP (Table 3). The LOO analysis indicates that the overall finding is not altered by removing any individual SNPs (Figure 6B), and the funnel plot is nearly symmetric (Figure 6C), meaning that our analyses are relatively well balanced.

### 3.5. Second MR Analysis of Se Levels with FN BMD, LS BMD, TB BMD and OP

After the first MR analysis was completed, we used the Phenoscanner V2 to check all SNPs to ensure that our analysis was valid. Surprisingly, two SNPs (rs6586282 and rs234709) were associated with homocysteine levels, whereas previous studies have shown that homocysteine levels are related to OP [53]. Next, rs6586282 and rs234709 were removed, and the causal association between Se concentrations and FN BMD, LS BMD, TB BMD and OP were analyzed again by MR. Unsurprisingly, the outcomes of the analysis remained consistent with the initial results even after the two SNPs were eliminated. The IVW method did not show a causal relationship between Se concentration and FN BMD (OR = 1.013, 95% CI = 0.985–1.042, *p* = 0.338), LS BMD (OR = 0.988, 95% CI = 0.957–1.020, *p* = 0.481), TB BMD (OR = 1.009, 95% CI = 0.988–1.030, *p* = 0.367) and OP (OR = 0.999, 95% CI = 0.998–1.001, *p* = 0.911) (Figure S1). Meanwhile, the results of the WM method (OR_FN BMD_ = 1.0.13, 95% CI_FN BMD_ = 0.976–1.051, *p*_FN BMD_ = 0.452; OR_LS BMD_ = 0.998, 95% CI_LS BMD_ = 0.958–1.040, *p*_LS BMD_ = 0.949; OR_TB BMD_ = 1.015, 95% CI_TB BMD_ = 0.989–1.041, *p*_TB BMD_ = 0.251; OR_OP_ = 0.999, 95% CI_OP_ = 0.998–1.001, *p*_OP_ = 0.796) and MR-Egger (OR_FN BMD_ = 1.068, 95% CI_FN BMD_ = 0.952–1.199, *p*_FN BMD_ = 0.293; OR_LS BMD_ = 1.034, 95% CI_LS BMD_ = 0.905–1.182, *p*_LS BMD_ = 0.629; OR_TB BMD_ = 1.012, 95% CI_TB BMD_ = 0.927–1.105, *p*_TB BMD_ = 0.786; OR_OP_ = 0.999, 95% CI_OP_ = 0.994–1.005, *p*_OP_ = 0.992) were in line with the IVW results (Figure S1).

### 3.6. Sensitivity Test of the Second MR Analysis

To test the validity of our analysis, we tested for heterogeneity and pleiotropy. The findings of the Cochran’s Q-test for MR-Egger (Q_FN BMD_ = 3.485, Q_LS BMD_ = 4.203, Q_TB BMD_ = 5.455, Q_OP_ = 13.276; *p*_FN BMD_ = 0.836, *p*_LS BMD_ = 0.756, *p*_TB BMD_ = 0.604, *p*_OP_ = 0.065) and IVW (Q_FN BMD_ = 4.355, Q_LS BMD_ = 4.683, Q_TB BMD_ = 5.460, Q_OP_ = 13.277; *p*_FN BMD_ = 0.823, *p*_LS BMD_ = 0.790, *p*_TB BMD_ = 0.707, *p*_OP_ = 0.102) were not statistically significant, indicating no heterogeneity (Table S1). The MR-Egger intercept results also suggest that our analysis has no pleiotropy (intercept_FN BMD_ = −0.012, intercept_LS BMD_ = −0.011, intercept_TB BMD_ = 0.000, intercept_OP_ = 0.000; *p*_FN BMD_ = 0.382, *p*_LS BMD_ = 0.510, *p*_TB BMD_ = 0.945, *p*_OP_ = 0.988) (Table S1). The LOO analysis revealed that no single SNP altered the overall result (Figure S2A–D). In addition, the funnel plot was approximately symmetric (Figure S3A–D), indicating the robustness of our results.

## 4. Discussion

Our investigation aimed to examine the correlation between selenium (Se) levels and the likelihood of developing osteoporosis (OP) through the utilization of Mendelian randomization (MR) analysis. Following the completion of two MR analyses, the Se levels showed no significant effect on the risk of OP in our findings.

Although there have been studies on the association between Se and OP, the findings have been inconsistent. Several studies have demonstrated that supplementation with Se does not have a significant impact on bone mineral density (BMD) and OP [31,32]. Similarly, Walsh et al. found that Se intake has no significant impact on bone improvement in postmenopausal women [54]. In contrast, Xie et al., reported that Se supplementation enhanced BMD and reduced the risk of OP [55], and a study by Park et al. found that low BMD was associated with decreased hair Se levels [56]. However, Xue et al. found that moderate Se intake increased BMD, whereas excessive intake reduced BMD [57]. Based on prior research, the association between Se and OP remains inconclusive and requires further investigation.

The reduction in BMD is one of the hallmarks of OP [7]. Therefore, in addition to analyzing the causal relationship between Se levels and OP, our study also analyzed femoral neck (FN) BMD, lumbar spine (LS) BMD and total body (TB) BMD to enhance the reliability of the results. Interestingly, Se levels were not related to either BMD or OP risk. The consistency of these results reinforces our view that Se levels have no effect on the risk of OP. We also found two SNPs (rs6586282 and rs234709) associated with homocysteine levels. Prior research has demonstrated that elevated levels of homocysteine are associated with an increased risk of OP and decreased BMD [53,58,59]. However, the findings of the two MR analyses conducted before and after the removal of rs6586282 and rs234709 were consistent. We believe that it may be the strong uncorrelation between Se and OP risk that is insufficient for confounding factors to alter the results. In addition, we also analyzed sensitivity and found no evidence of heterogeneity or pleiotropy of single-nucleotide polymorphisms (SNPs). This further suggests that our findings are stable and reliable. Although a causal association between Se and OP risk was not found in our study, whether Se may indirectly affect OP through other pathways needs to be further investigated.

It is known that the current study is the first MR study to assess the impact of Se levels on OP risk, explaining whether there is a causal association between Se levels and the risk of OP at the genetic level. MRs are simulated randomized controlled trials by means of IVs, and they have great advantages [60]. Reverse causality can be avoided because the IVs associated with exposure are determined before birth [61]. When MR analysis is performed, confounding IVs are excluded, which leads to more reliable results [62]. MR analysis provides more evidence and effectively avoids bias [63].

Our study, however, has some limitations. First, the focus of this study was confined to a European population as the MR was performed mainly on a European sample, thereby creating uncertainty regarding the generalizability of the results to populations worldwide. Hence, our results need further validation in diverse populations across various geographical regions. Second, due to the utilization of a publicly available GWAS database, it was unfeasible to assess potential sample overlap. Third, the 11 SNPs used in our investigation are instrumental variables from two loci only (chr5 and chr21), and maybe, in the future, larger GWAS studies will identify more associated SNPs, increasing the power of MR. Finally, our focus was solely on examining the association between levels of Se in toenails and blood and the risk of OP. However, it is important to note that the potential impact of Se levels in other bodily tissues and overall Se status on OP risk remains an area that requires additional research and investigation.

## 5. Conclusions

To conclude, our study has determined that there is no sufficient evidence to support a causal association between selenium (Se) levels and the likelihood of developing osteoporosis (OP). Therefore, it is suggested that the use of Se supplementation as a preventive measure against OP may not be an effective approach. Given the lack of substantial evidence supporting the significant effect of Se on the risk of OP in this context, it is therefore concluded that this area of research needs to be further explored.

## Figures and Tables

**Table 1:** The 11 SNPs associated with selenium levels.

SNP	Chromosome	Effect Allele	Other Allele	Frequency	Beta	Se	P
rs672413	5	A	G	0.32	0.164418	0.021835	5.21 × 10^−14^
rs705415	5	T	C	0.14	−0.20006	0.032113	4.64 × 10^−10^
rs3797535	5	T	C	0.08	0.298102	0.037544	2.05 × 10^−15^
rs11951068	5	A	G	0.07	0.268264	0.03992	1.86 × 10^−11^
rs921943	5	T	C	0.29	0.294952	0.022447	1.90 × 10^−39^
rs10944	5	T	G	0.49	0.257746	0.020375	1.13 × 10^−36^
rs567754	5	T	C	0.34	−0.19588	0.021502	8.38 × 10^−20^
rs6859667	5	T	C	0.96	−0.35969	0.051978	4.40 × 10^−12^
rs6586282	21	T	C	0.17	−0.15971	0.027116	3.96 × 10^−9^
rs1789953	21	T	C	0.14	0.162035	0.029354	3.40 × 10^−8^
rs234709	21	T	C	0.45	−0.11957	0.020474	5.23 × 10^−9^

**Table 2:** GWASs information is incorporated into the Mendelian randomization.

GWAS ID	Trait	Sample Size	Number of SNPs	Population	PMID
ieu-a-980	FN BMD	32,735	10,586,900	European	26367794
ieu-a-982	LS BMD	28,498	10,582,867	European	26367794
ebi-a-GCST005348	TB BMD	56,284	16,162,733	European	29304378
ebi-a-GCST90038656	OP	484,598	9,587,836	European	33959723

**Table 3:** Heterogeneity and pleiotropy test.

Exposure	Outcome	Heterogeneity Test MR–Egger		Heterogeneity Test IVW		Pleiotropy Test MR–Egger	
		Q	P	Q	P	Intercept	P
Se levels	FN BMD	4.919	0.841	9.058	0.526	−0.020	0.072
Se levels	LS BMD	4.827	0.849	7.574	0.670	−0.018	0.131
Se levels	TB BMD	6.460	0.693	7.040	0.721	−0.005	0.465
Se levels	OP	14.772	0.097	16.063	0.097	0.000	0.398

## Data Availability

The data supporting the findings of this study are available on the IEU open GWAS project websites (https://gwas.mrcieu.ac.uk/, accessed on 18 October 2023).

## Supplementary Materials

The following supporting information can be downloaded at: https://www.mdpi.com/article/10.3390/nu15245065/s1, Figure S1: Odds ratio plot of selenium levels with OP and BMD from the second MR analysis; Figure S2: Forest plots of leave-one-out analyses of the causal effect of selenium levels on OP and BMD from the second MR analysis; Figure S3: Funnel plots of the causal effect of selenium levels on OP and BMD from the second MR analysis; Table S1: Heterogeneity and pleiotropy test from the second MR analysis.

## References

[1] Levy G., Levin B. (2022). An Evolution-Based Model of Causation for Aging-Related Diseases and Intrinsic Mortality: Explanatory Properties and Implications for Healthy Aging. Front. Public Health.

[2] Schrempft S., Belsky D.W., Draganski B., Kliegel M., Vollenweider P., Marques-Vidal P., Preisig M., Stringhini S. (2022). Associations between Life-Course Socioeconomic Conditions and the Pace of Aging. J. Gerontol. Ser. A-Biol. Sci. Med. Sci..

[3] Looker A.C., Isfahani N.S., Fan B., Shepherd J.A. (2017). Trends in osteoporosis and low bone mass in older US adults, 2005–2006 through 2013–2014. Osteoporos. Int..

[4] Wright N.C., Looker A.C., Saag K.G., Curtis J.R., Delzell E.S., Randall S., Dawson-Hughes B. (2014). The Recent Prevalence of Osteoporosis and Low Bone Mass in the United States Based on Bone Mineral Density at the Femoral Neck or Lumbar Spine. J. Bone Miner. Res..

[5] (2001). NIH Consensus Development Panel on Osteoporosis Prevention, Diagnosis, and Therapy, March 7–29, 2000: Highlights of the conference. South. Med. J..

[6] Osnes E.K., Lofthus C.M., Meyer H.E., Falch J.A., Nordsletten L., Cappelen I., Kristiansen I.S. (2004). Consequences of hip fracture on activities of daily life and residential needs. Osteoporos. Int..

[7] Liu J., Curtis E.M., Cooper C., Harvey N.C. (2019). State of the art in osteoporosis risk assessment and treatment. J. Endocrinol. Investig..

[8] Leder B.Z., Mitlak B., Hu M.-Y., Hattersley G., Bockman R.S. (2020). Effect of Abaloparatide vs Alendronate on Fracture Risk Reduction in Postmenopausal Women with Osteoporosis. J. Clin. Endocrinol. Metab..

[9] Black D.M., Geiger E.J., Eastell R., Vittinghoff E., Li B.H., Ryan D.S., Dell R.M., Adams A.L. (2020). Atypical Femur Fracture Risk versus Fragility Fracture Prevention with Bisphosphonates. N. Engl. J. Med..

[10] Xu X.-H., Dong S.-S., Guo Y., Yang T.-L., Lei S.-F., Papasian C.J., Zhao M., Deng H.-W. (2010). Molecular Genetic Studies of Gene Identification for Osteoporosis: The 2009 Update. Endocr. Rev..

[11] Wang L., Ran L., Zha X., Zhao K., Yang Y., Shuang Q., Liu Y., Hind K., Cheng X., Blake G.M. (2020). Adjustment of DXA BMD measurements for anthropometric factors and its impact on the diagnosis of osteoporosis. Arch. Osteoporos..

[12] Hirschfeld H.P., Kinsella R., Duque G. (2017). Osteosarcopenia: Where bone, muscle, and fat collide. Osteoporos. Int..

[13] Ralston S.H., Uitterlinden A.G. (2010). Genetics of Osteoporosis. Endocr. Rev..

[14] Brommage R., Ohlsson C. (2018). Translational studies provide insights for the etiology and treatment of cortical bone osteoporosis. Best Pract. Res. Clin. Endocrinol. Metab..

[15] Wilson M.L., Fleming K.A., Kuti M.A., Looi L.M., Lago N., Ru K. (2018). Pathology and laboratory medicine in low-income and middle-income countries 1: Access to pathology and laboratory medicine services: A crucial gap. Lancet.

[16] Burk R.F., Hill K.E., Motley A.K., Winfrey V.P., Kurokawa S., Mitchell S.L., Zhang W. (2014). Selenoprotein P and apolipoprotein E receptor-2 interact at the blood-brain barrier and also within the brain to maintain an essential selenium pool that protects against neurodegeneration. FASEB J..

[17] Bjorklund G., Shanaida M., Lysiuk R., Antonyak H., Klishch I., Shanaida V., Peana M. (2022). Selenium: An Antioxidant with a Critical Role in Anti-Aging. Molecules.

[18] Radomska D., Czarnomysy R., Radomski D., Bielawska A., Bielawski K. (2021). Selenium as a Bioactive Micronutrient in the Human Diet and Its Cancer Chemopreventive Activity. Nutrients.

[19] Hariharan S., Dharmaraj S. (2020). Selenium and selenoproteins: It’s role in regulation of inflammation. Inflammopharmacology.

[20] Nicholson J.L., Toh P., Alfulaij N., Berry M.J., Torres D.J. (2022). New insights on selenoproteins and neuronal function. Free Radic. Biol. Med..

[21] Arbogast S., Ferreiro A. (2010). Selenoproteins and Protection against Oxidative Stress: Selenoprotein N as a Novel Player at the Crossroads of Redox Signaling and Calcium Homeostasis. Antioxid. Redox Signal..

[22] Gao X., Ye C., Ma H., Zhang Z., Wang J., Zhang Z.-H., Zhao X., Ho C.-T. (2023). Research Advances in Preparation, Stability, Application, and Possible Risks of Nanoselenium: Focus on Food and Food-Related Fields. J. Agric. Food Chem..

[23] Johnson L.A., Phillips J.A., Mauer C., Edwards M., Balldin V.H., Hall J.R., Barber R., Conger T.L., Ho E.J., O’Bryant S.E. (2013). The impact of GPX1 on the association of groundwater selenium and depression: A project FRONTIER study. BMC Psychiatry.

[24] Flores-Mateo G., Navas-Acien A., Pastor-Barriuso R., Guallar E. (2006). Selenium and coronary heart disease: A meta-analysis. Am. J. Clin. Nutr..

[25] Hu X.F., Stranges S., Chan L.H.M. (2019). Circulating Selenium Concentration Is Inversely Associated with the Prevalence of Stroke: Results from the Canadian Health Measures Survey and the National Health and Nutrition Examination Survey. J. Am. Heart Assoc..

[26] Stranges S., Marshall J.R., Natarajan R., Donahue R.P., Trevisan M., Combs G.F., Cappuccio F.P., Ceriello A., Reid M.E. (2007). Effects of long-term selenium supplementation on the incidence of type 2 diabetes—A randomized trial. Ann. Intern. Med..

[27] Grili P.P.d.F., Vidigal C.V., da Cruz G.F., Albergaria B.H., Marques-Rocha J.L., Pereira T.S.S., Guandalini V.R. (2022). Dietary consumption of selenium inversely associated with osteoporosis in postmenopausal women. Front. Nutr..

[28] Peng S., Zhang G., Wang D. (2023). Association of selenium intake with bone mineral density and osteoporosis: The national health and nutrition examination survey. Front. Endocrinol..

[29] Zhang J.J., Munger R.G., West N.A., Cutler D.R., Wengreen H.J., Corcoran C.D. (2006). Antioxidant intake and risk of osteoporotic hip fracture in Utah: An effect modified by smoking status. Am. J. Epidemiol..

[30] Arikan D.C., Coskun A., Ozer A., Kilinc M., Atalay F., Arikan T. (2011). Plasma Selenium, Zinc, Copper and Lipid Levels in Postmenopausal Turkish Women and Their Relation with Osteoporosis. Biol. Trace Elem. Res..

[31] Ilich J.Z., Cvijetic S., Baric I.C., Cecic I., Saric M., Crncevic-Orlic Z., Blanusa M., Korsic M. (2009). Nutrition and lifestyle in relation to bone health and body weight in Croatian postmenopausal women. Int. J. Food Sci. Nutr..

[32] Odabasi E., Turan M., Aydin A., Akay C., Kutlu M. (2008). Magnesium, zinc, copper, manganese, and selenium levels in postmenopausal women with osteoporosis. Can magnesium play a key role in osteoporosis?. Ann. Acad. Med. Singap..

[33] Smith G.D., Ebrahim S. (2003). ‘Mendelian randomization’: Can genetic epidemiology contribute to understanding environmental determinants of disease?. Int. J. Epidemiol..

[34] Sanderson E., Glymour M.M., Holmes M.V., Kang H., Morrison J., Munafo M.R., Palmer T., Schooling C.M., Wallace C., Zhao Q. (2022). Mendelian randomization. Nat. Rev. Methods Primers.

[35] Lawlor D.A., Harbord R.M., Sterne J.A.C., Timpson N., Smith G.D. (2008). Mendelian randomization: Using genes as instruments for making causal inferences in epidemiology. Stat. Med..

[36] Holmes M.V., Ala-Korpela M., Smith G.D. (2017). Mendelian randomization in cardiometabolic disease: Challenges in evaluating causality. Nat. Rev. Cardiol..

[37] Emdin C.A., Khera A.V., Kathiresan S. (2017). Mendelian Randomization. JAMA-J. Am. Med. Assoc..

[38] Cheng L., Zhuang H., Yang S., Jiang H., Wang S., Zhang J. (2018). Exposing the Causal Effect of C-Reactive Protein on the Risk of Type 2 Diabetes Mellitus: A Mendelian Randomization Study. Front. Genet..

[39] Zhuang H., Han J., Cheng L., Liu S.-L. (2019). A Positive Causal Influence of IL-18 Levels on the Risk of T2DM: A Mendelian Randomization Study. Front. Genet..

[40] Chen X., Kong J., Pan J., Huang K., Zhou W., Diao X., Cai J., Zheng J., Yang X., Xie W. (2021). Kidney damage causally affects the brain cortical structure: A Mendelian randomization study. Ebiomedicine.

[41] Cornelis M.C., Fornage M., Foy M., Xun P., Gladyshev V.N., Morris S., Chasman D.I., Hu F.B., Rimm E.B., Kraft P. (2015). Genome-wide association study of selenium concentrations. Hum. Mol. Genet..

[42] Evans D.M., Zhu G., Dy V., Heath A.C., Madden P.A.F., Kemp J.P., McMahon G., St Pourcain B., Timpson N.J., Golding J. (2013). Genome-wide association study identifies loci affecting blood copper, selenium and zinc. Hum. Mol. Genet..

[43] Fu S., Zhang L., Ma F., Xue S., Sun T., Xu Z. (2022). Effects of Selenium on Chronic Kidney Disease: A Mendelian Randomization Study. Nutrients.

[44] Burgess S., Thompson S.G. (2011). Bias in causal estimates from Mendelian randomization studies with weak instruments. Stat. Med..

[45] Zheng H.-F., Forgetta V., Hsu Y.-H., Estrada K., Rosello-Diez A., Leo P.J., Dahia C.L., Park-Min K.H., Tobias J.H., Kooperberg C. (2015). Whole-genome sequencing identifies EN1 as a determinant of bone density and fracture. Nature.

[46] Medina-Gomez C., Kemp J.P., Trajanoska K., Luan J.a., Chesi A., Ahluwalia T.S., Mook-Kanamori D.O., Ham A., Hartwig F.P., Evans D.S. (2018). Life-Course Genome-wide Association Study Meta-analysis of Total Body BMD and Assessment of Age-Specific Effects. Am. J. Hum. Genet..

[47] Donertas H.M., Fabian D.K., Fuentealba M., Partridge L., Thornton J.M. (2021). Common genetic associations between age-related diseases. Nat. Aging.

[48] Bowden J., Smith G.D., Burgess S. (2015). Mendelian randomization with invalid instruments: Effect estimation and bias detection through Egger regression. Int. J. Epidemiol..

[49] Grover S., Del Greco M.F., Stein C.M., Ziegler A. (2017). Mendelian Randomization. Methods Mol. Biol..

[50] Chen X., Kong J., Diao X., Cai J., Zheng J., Xie W., Qin H., Huang J., Lin T. (2020). Depression and prostate cancer risk: A Mendelian randomization study. Cancer Med..

[51] Burgess S., Thompson S.G. (2017). Interpreting findings from Mendelian randomization using the MR-Egger method. Eur. J. Epidemiol..

[52] Gronau Q.F., Wagenmakers E.-J. (2019). Limitations of Bayesian Leave-One-Out Cross-Validation for Model Selection. Comput. Brain Behav..

[53] Herrmann M., Widmann T., Herrmann W. (2005). Homocysteine—A newly recognised risk factor for osteoporosis. Clin. Chem. Lab. Med..

[54] Walsh J.S., Jacques R.M., Schomburg L., Hill T.R., Mathers J.C., Williams G.R., Eastell R. (2021). Effect of selenium supplementation on musculoskeletal health in older women: A randomised, double-blind, placebo-controlled trial. Lancet Healthy Longev..

[55] Xie H., Wang N., He H., Yang Z., Wu J., Yang T., Wang Y. (2023). The association between selenium and bone health: A meta-analysis. Bone Jt. Res..

[56] Park K.-C., Kwon Y., Lee Y., Kim D.K., Jang Y., Lee S. (2020). Low selenium levels are associated with decreased bone mineral densities. J. Trace Elem. Med. Biol..

[57] Xue G., Liu R. (2022). Association between dietary selenium intake and bone mineral density in the US general population. Ann. Transl. Med..

[58] Bailey R.L., Looker A.C., Lu Z.H., Fan R.Z., Eicher-Miller H.A., Fakhouri T.H., Gahche J.J., Weaver C.M., Mills J.L. (2015). B-vitamin status and bone mineral density and risk of lumbar osteoporosis in older females in the United States. Am. J. Clin. Nutr..

[59] Bucciarelli P., Martini G., Martinelli I., Ceccarelli E., Gennari L., Bader R., Valenti R., Franci B., Nuti R., Mannucci P.M. (2010). The relationship between plasma homocysteine levels and bone mineral density in post-menopausal women. Eur. J. Intern. Med..

[60] Davey Smith G., Hemani G. (2014). Mendelian randomization: Genetic anchors for causal inference in epidemiological studies. Hum. Mol. Genet..

[61] Qian Y., Ye D., Huang H., Wu D.J.H., Zhuang Y., Jiang X., Mao Y. (2020). Coffee Consumption and Risk of Stroke: A Mendelian Randomization Study. Ann. Neurol..

[62] Robinson P.C., Choi H.K., Do R., Merriman T.R. (2016). Insight into rheumatological cause and effect through the use of Mendelian randomization. Nat. Rev. Rheumatol..

[63] VanderWeele T.J., Tchetgen E.J.T., Cornelis M., Kraft P. (2014). Methodological Challenges in Mendelian Randomization. Epidemiology.

