# Supplementary material for: Causal Effect of Selenium Levels on Osteoporosis: A Mendelian Randomization Study
Source: Nutrients. 2023 Dec 11;15(24):5065. doi: 10.3390/nu15245065 (PMC10746097; doi:10.3390/nu15245065)
Supplement: Supplementary file 1 [file nutrients-15-05065-s001.zip › Supplementary information.pdf]

Contents

Supplementary Figure S1. Odds ratio plot of selenium levels with OP and BMD from the second MR analysis.

Supplementary Figure S2. Forest plots of leave-one-out analyses of the causal effect of selenium levels on OP and BMD from the second MR analysis.

Supplementary Figure S3. Funnel plots of the causal effect of selenium levels on OP and BMD from the second MR analysis.

Supplementary Table S1. Heterogeneity and pleiotropy test from the second MR analysis.

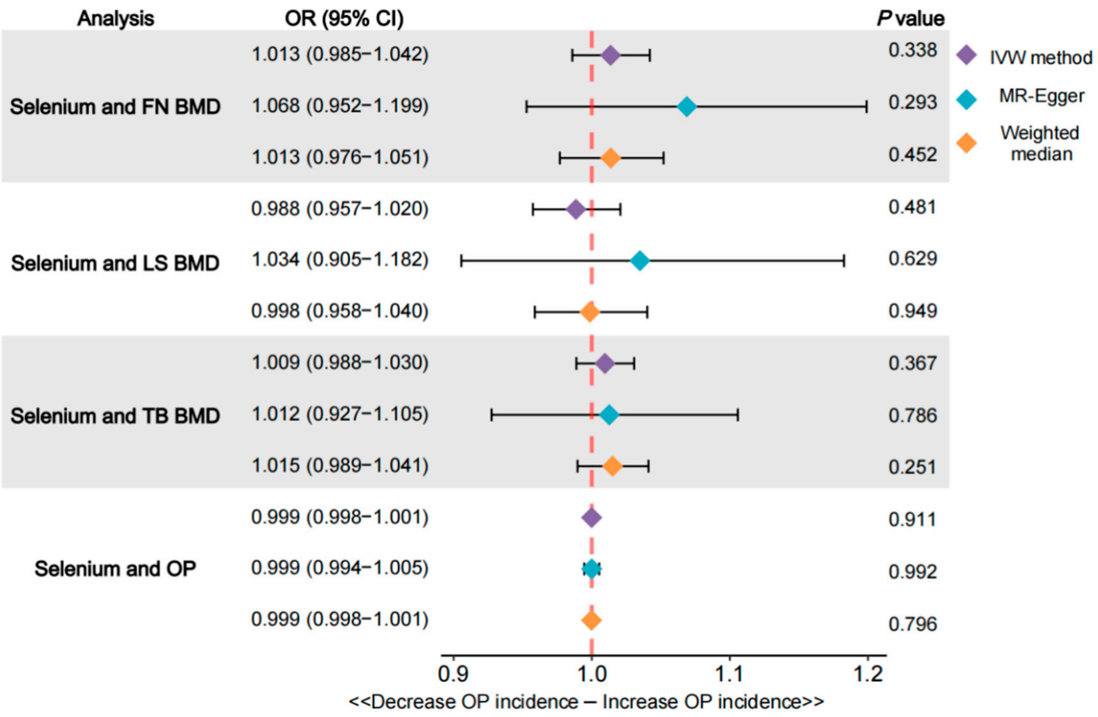

Supplementary Figure S1. Odds ratio plot of selenium levels with OP and BMD from the second MR analysis. OR, odds ratio; FN BMD, femoral neck bone mineral density; LS BMD, lumbar spine bone mineral density; TB BMD, total body bone mineral density; OP, osteoporosis; IVW, inverse variance weighted.

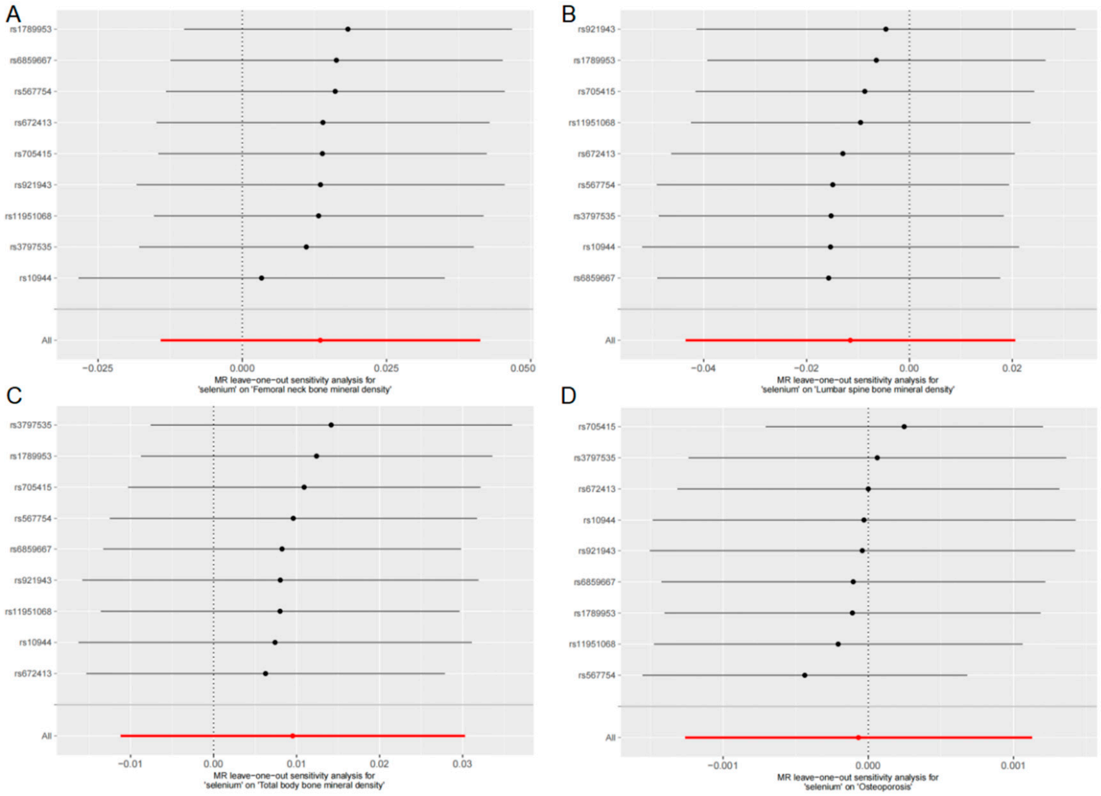

Supplementary Figure S2. Forest plots of leave-one-out analyses of the causal effect of selenium levels on OP and BMD from the second MR analysis (A) FN BMD; (B) LS BMD; (C) TB BMD; (D) OP.

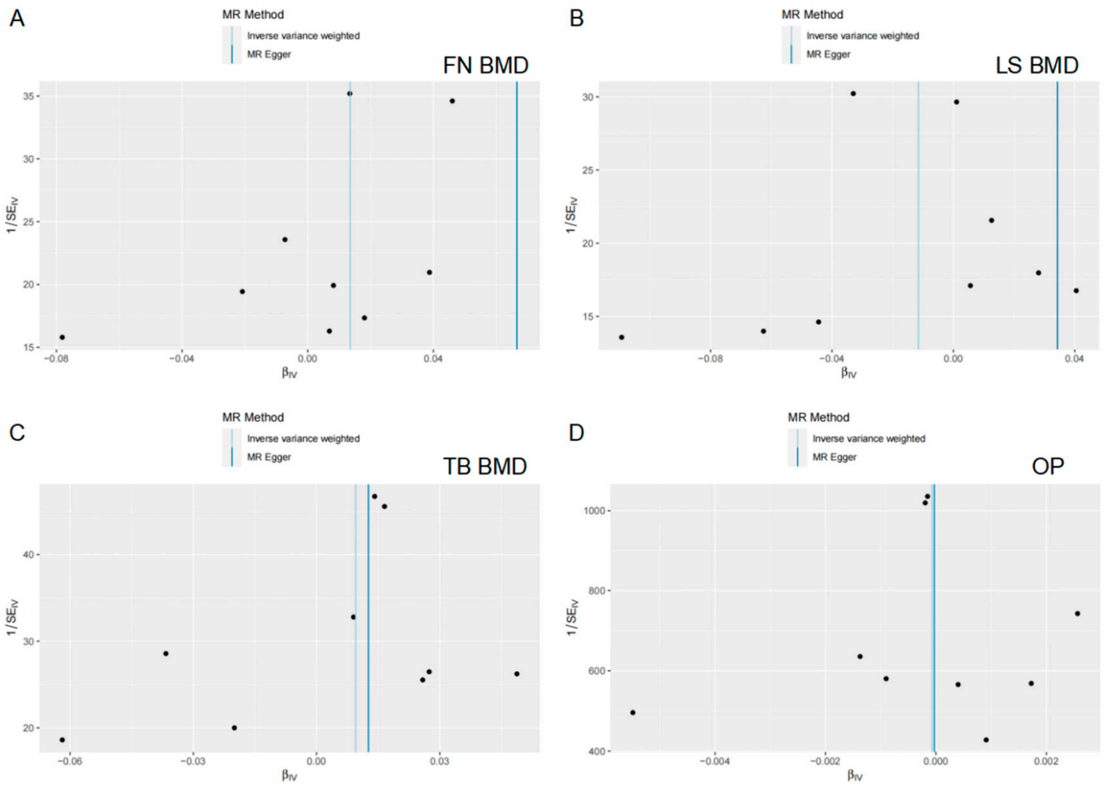

Supplementary Figure S3. Funnel plots of the causal effect of selenium levels on OP and BMD from the second MR analysis (A) FN BMD; (B) LS BMD; (C) TB BMD; (D) OP.

Exposure	Outcome	Heterogeneity test MR Egger		Heterogeneity test IVW		Pleiotropy test MR Egger	
		Q	P	Q	P	Intercept	P
Se levels	FN BMD	3.485	0.836	4.355	0.823	-0.012	0.382
Se levels	LS BMD	4.203	0.756	4.683	0.790	-0.011	0.510
Se levels	TB BMD	5.455	0.604	5.460	0.707	0.000	0.945
Se levels	OP	13.276	0.065	13.277	0.102	0.000	0.988

Supplementary Table S1. Heterogeneity and pleiotropy test from the second MR analysis.
